# Predictors for second-stage posterior direct decompression after lateral lumbar interbody fusion: a review of five hundred fifty-seven patients in the past five years

**DOI:** 10.1007/s00264-022-05313-4

**Published:** 2022-02-07

**Authors:** Jun Li, Tian-zhen Xu, Ning Zhang, Qi-xin Chen, Fang-cai Li

**Affiliations:** 1 Department of Orthopedics, The Second Affiliated Hospital, School of Medicine, Zhejiang University, No.88 Jiefang Road, Hangzhou, 310009 Zhejiang Province China; 2 Department of Orthopedics, Zhuji People’s Hospital of Zhejiang Province, Shaoxing, Zhejiang Province, China

**Keywords:** Lateral lumbar interbody fusion, Indirect decompression, Lumbar spinal stenosis, Central canal, Facet joint, Lateral recess, Foramen

## Abstract

**Purpose:**

To analyze the predictors for second-stage posterior direct decompression (PDD) after lateral lumbar interbody fusion (LLIF) procedure.

**Methods:**

We studied patients who underwent LLIF for degenerative lumbar spinal stenosis in the last five years, from July 2016 to June 2021. All surgical levels were grouped according to Schizas’ central canal stenosis (CCS) classification, Pathria’s facet joint degeneration (FJD) classification, Bartynski’s lateral recess stenosis (LRS) classification, and Lee’s foraminal stenosis (FS) classification. Second-stage PDD rates of each subgroup and their annual change were analyzed. Evaluation of risk factors associated with PDD was investigated.

**Results:**

A total of 901 segments from 557 patients were included. The overall PDD rate was 29.97%. An overall PDD rate of 75.21% for grade D CCS, 29.74% for grade C CCS, 41.67% for grade 3 FJD, 37.61% for grade 3 LRS, and 40.70% for grade 3 FS was shown. While there was a continuous decline in annual PDD rate in the past four years, the annual PDD rate for grade D remained at very high levels. Logistic regression analysis had shown grade D CCS as the utmost risk factor for PDD (OR = 17.77). And grade 3 LRS (OR = 4.63), grade 3 FS (OR = 2.42), grade C CCS (OR = 2.41), and grade 3 FJD (OR = 2.04) were also moderately correlated with PDD, which meant they only moderately increased the risk of PDD.

**Conclusion:**

Extreme severe lumbar CCS (grade D) is the greatest determinant to perform the second-stage PDD procedure after LLIF.

## Introduction

The minimally invasive lateral lumbar interbody fusion (LLIF) is an indirect decompression technique, resulting in a lower approach-related morbidity in comparison to traditional open decompression techniques [1, 2]. Radiographic decompression results reveal LLIF to be significantly worse when compared to minimally invasive transforaminal lumbar interbody fusion (MIS-TLIF) [3], let alone conventional TLIF or posterior lumbar interbody fusion. Although plenty of studies had previously shown that the clinical outcome of LLIF for LSS is comparable to MIS-TLIF and TLIF [4–9], a surgical indication of LLIF for LSS varied amongst different surgeons. It is noted that there are still doubts or hesitance in performing the LLIF procedures on patients with severe lumbar degenerative diseases.

According to current research, the possible risk factor of failed indirect decompression includes severe central canal stenosis (CCS) [10–12], bony lateral recess stenosis (LRS) [13], uncontained disc herniation, locked facet, severe hypertrophy of ligamentum flavum [14], cage subsidence [15, 16], and osteoporosis [17]. In clinical settings, due to poorly recognized indications to perform PDD, nor the definition regarding indirect decompression failure, additional PDD rate after LLIF showed variation across different studies with a range of 0 ~ 72% [13, 15, 16, 18–23]. Many of those PDD were likely unnecessary. According to these studies, we should include the risk factors for PDD in further studies to determine a clear indication of LLIF for lumbar spinal stenosis (LSS).

In this study, we retrospectively studied patients who underwent LLIF for LSS over the last five years in our institution, where an annual change of PDD rate data was collected. PDD rates obtained were further compared between subgroups according to different radiographic classifications. We then analyzed the risk factors for PDD through univariate and multivariable logistic regression model, which helped us better acknowledge the indication of LLIF for LSS.

## Materials and methods

### Patients

In this retrospective study, patients with the main diagnosis of degenerative LSS had undergone a procedure performed by our surgical group between July 2016 and June 2021. The procedure involved was crenel lateral lumbar interbody fusion (CLIF, SANYOU, China), which is a modified extreme lateral interbody fusion technique (XLIF). Patients who suffered from significant lumbar scoliosis (Cobb ≥ 20°), grade two spondylolisthesis, and lumbar fracture or had undergone prior lumbar surgery were excluded from this study.

### Evaluation

**Fig. 1:**
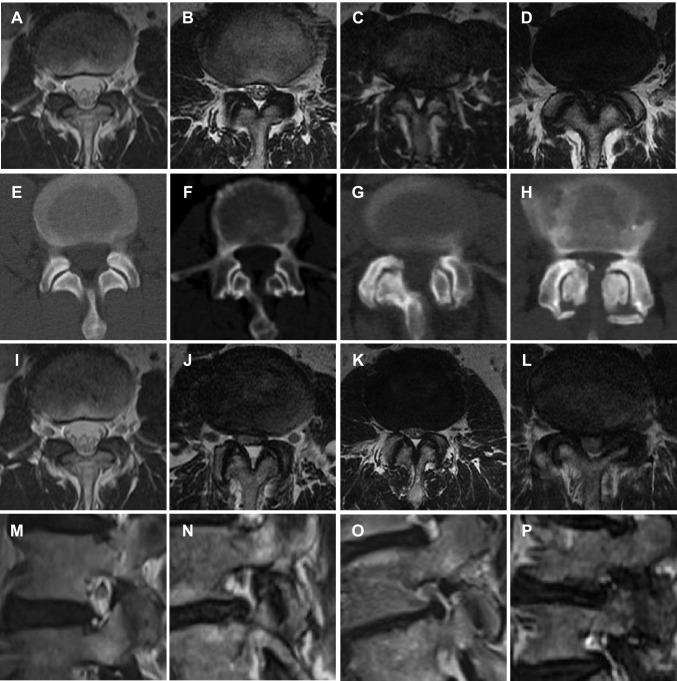
Typical images illustrate radiographic classifications. Schizas’ central canal stenosis (CCS) classification (**a**–**d**), **a** Grade A; **b** Grade B; **c** Grade C; **d** Grade D. Pathria’s facet joint degeneration (FJD) classification (**e**–**h**), **e** Grade 0; **f** Grade 1; **g** Grade 2; **h** Grade 3. Bartynski’s lateral recess stenosis (LRS) classification (**i**–**l**), **i** Grade 0; **j** Grade 1; **k** Grade 2; **l** Grade 3. Lee’s foraminal stenosis (FS) classification (**m**–**p**), **m** Grade 0; **n** Grade 1; **o** Grade 2; **p** Grade 3

Demographic data were collected. The annual PDD rate, staged surgery rate, average hospital stays, and average cage height of consecutive five years, namely from July 2016 to June 2017, July 2017 to June 2018, July 2018 to June 2019, July 2019 to June 2020, and July 2020 to June 2021, were collected. All these segments were then further grouped according to Schizas’ CCS classification [24], Bartynski’s LRS classification [25] and Lee’s foraminal stenosis (FS) classification [26] on MRI, and Pathria’s facet joint degeneration (FJD) classification [27] on CT (Fig. 1). CCS were graded as A (mild), B (moderate), C (severe), and D (extremely severe). LRS, FS, and FJD were graded as 0 (normal), 1 (mild), 2 (moderate), and 3 (severe). FJD, LRS, and FS should have been evaluated on both sides. However, we had only accounted for the severe side. The radiographic measurements were performed by board-certified spine surgery fellows (T.X and J.L.). PDD rate of each subgroup and their annual change were further compared.

### Surgical techniques

The CLIF technique is a modified technique of XLIF, aimed to minimize approach-related complications. For patients with staged surgery, posterior instrumentation with/without PDD is performed one week after the first stage. When there is an inadequate resolution of stenotic symptoms or radicular leg pain, and a positive straight leg raise test or femoral nerve stretch test after CLIF, PDD will be performed. The indication of PDD may vary amongst different attending surgeons, where views on the matter may have slightly changed in the past five years.

### Statistics

Descriptive data are represented as means ± standard deviation (SD). The pre-operative variables can contribute to PDD including age, sex, BMI, diagnosis, surgical segments, cage height, and radiographic parameters, which were first analyzed by the univariate correlation. Categorical variables were presented as numbers and percentages (%). A chi-squared test was used in the analysis of categorical variables. Variables that were significantly associated with PDD in the univariate analysis were entered into a multivariable logistic regression model to identify the independent pre-operative radiologic factors predictive of PDD. In multivariable logistic regression model, we adjusted for age, sex, BMI, cage height, and number of surgical segments and whether there was spondylolisthesis as contributors. To further explore the effect of surgical experience on results, a subgroup analysis was conducted according to different years. A two-sided *P* value < 0.05 was deemed statistically significant. All statistical analyses were performed using R (version 4.1.0; R Development Core Team).

## Results

A total of 901 segments from 557 patients who underwent LLIF were studied, which includes one segment of L1/2, 85 segments of L2/3, 306 segments of L3/4, and 509 segments of L4/5. The mean age of patients was 65.22 ± 7.64 years, and the mean BMI was 24.66 ± 3.18 kg/m^2^. The patient sample group consists of 260 males and 297 females. The mean cage height was 12.97 ± 1.44 mm. One-level fusion was performed in 278 patients, two-level fusion in 214 patients, and three-level fusion in 65 patients. Spondylolisthesis was observed at 31.85% (287/901) of all surgical segments.

Table 1 displays a trend, where annual surgical procedures and surgical segments were observed to have been increasing in the last five years, whereas staged surgery rates were seen decreasing. This is attributed to our improvement in understanding indications for LLIF in LSS. Due to fewer staged surgical procedures, average hospital stays were continuously decreasing in the past five years. The overall PDD rate is 29.97% (270/901). The annual PDD rate observed a continuous decline in the past four years, and most recently showed a rate of 23.13% in the last year.

**Table 1:** Annual change of parameters related with LLIF surgery for LSS

Year	Surgeries (*n*)	Staged surgery	Staged surgery rate (%)	Segments (*n*)	PDD (*n*)	PDD rate (%)	Hospital stay (days)	Cage height (mm)
2016–2017	39	32	82.05 (32/39)	65	15	23.78 (15/65)	18.21 ± 4.45	13.40 ± 1.37
2017–2018	99	89	89.90 (89/89)	145	62	42.76 (62/145)	18.45 ± 6.07	13.45 ± 1.20
2018–2019	120	97	80.83 (97/120)	193	67	34.72 (67/193)	17.61 ± 6.34	13.43 ± 1.26
2019–2020	131	79	60.31 (79/131)	204	58	28.43 (58/204)	13.84 ± 5.60	12.95 ± 1.41
2020–2021	168	91	54.17 (91/168)	294	68	23.13 (68/294)	12.04 ± 5.00	12.34 ± 1.46

*LLIF* lateral lumbar interbody fusion; *LSS* lumbar spinal stenosis; *PDD* posterior direct decompression

**Fig. 2:**
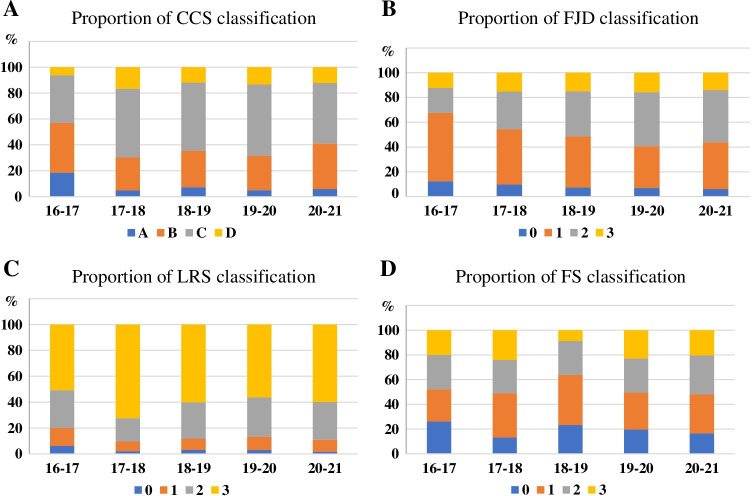
Annual proportion according to radiographic classification. **a** Central canal stenosis (CCS) classification; **b** facet joint degeneration (FJD) classification; **c** lateral recess stenosis (LRS) classification; **d** foraminal stenosis (FS) classification

A significant trend observed for CCS, FJD, LRS, and FS is where there are more severe grading classifications, the higher the total PDD rate (Table 2). However, there were no significant differences in the proportion of four grades in those parameters in the past 5 years (Fig. 2). CCS grade C or D segments account for 63.04% (568/901) of all segments. FJD grade 3 segments account for 14.65% (132/901). LRS grade 3 segments account for 60.49% (545/901). FS grade 3 segments account for 19.09% (172/901).

**Table 2:** Annual change of PDD rate according to radiographic classifications

	Total PDD rate (%)	2016–2017 PDD rate (%)	2017–2018 PDD rate (%)	2018–2019 PDD rate (%)	2019–2020 PDD rate (%)	2020–2021 PDD rate (%)
CCS grade						
A	16.67% (10/60)	8.33% (1/12)	14.29% (1/7)	28.57% (4/14)	30% (3/10)	5.88% (1/17)
B	14.65% (40/273)	20% (5/25)	21.62% (8/37)	11.11% (6/54)	7.41% (4/54)	16.50% (17/103)
C	29.74% (135/454)	25% (6/24)	46.75% (36/77)	37.25% (38/102)	27.43% (31/113)	17.39% (24/138)
D	74.56% (85/114)	75% (3/4)	70.83% (17/24)	82.61% (19/23)	74.07% (20/27)	72.22% (26/36)
FJD grade						
0	13.24% (9/68)	1.25% (1/8)	14.29% (2/14)	21.43% (3/14)	7.14% (1/14)	11.11% (2/18)
1	28.25% (102/361)	22.22% (8/36)	35.38% (23/65)	30% (24/80)	26.09% (18/69)	26.13% (29/111)
2	30.59% (104/340)	30.77% (4/13)	50% (22/44)	40% (28/70)	29.21% (26/89)	19.35% (24/124)
3	41.67% (55/132)	25% (2/8)	68.18% (15/22)	41.38% (12/29)	40.63% (13/32)	31.71% (13/41)
LRS grade						
0	10% (2/20)	0% (0/4)	0% (0/3)	16.67% (1/6)	16.67% (1/6)	0% (0/5)
1	12.36% (11/89)	22.22% (2/9)	18.18% (2/11)	5.89% (1/17)	14.29% (3/21)	11.11% (3/27)
2	20.97% (52/248)	10.53% (2/19)	26.92% (7/26)	27.78% (15/54)	19.35% (12/62)	18.60% (16/86)
3	37.61% (205/545)	33.33% (11/33)	50.48% (53/105)	43.10% (50/116)	36.52% (42/115)	27.84% (49/176)
FS grade						
0	17.06% (29/170)	23.53% (4/17)	21.05% (4/19)	20% (9/45)	17.50% (7/40)	10.20% (5/49)
1	26.33% (79/300)	17.65% (3/17)	18.18% (19/52)	34.62% (27/78)	18.03% (11/61)	20.65% (19/92)
2	35.52% (92/259)	22.22% (4/18)	46.15% (18/39)	43.40% (23/53)	35.71% (20/56)	29.03% (27/93)
3	40.70% (70/172)	30.77% (4/13)	60% (21/35)	47.06% (8/17)	42.55% (20/47)	28.33% (17/60)
Diagnosis						
LSS	28.62% (176/615)	27.5% (11/40)	33.33% (27/81)	35.88% (47/131)	30.99% (44/142)	21.36% (47/220)
LSS/spondylolisthesis	33.94% (94/277)	16% (4/25)	54.69% (35/64)	32.26% (20/62)	22.58% (14/62)	28.38% (21/74)

*CCS* central canal stenosis; *FJD* facet joint degeneration; *LRS* lateral recess stenosis; *FS* foraminal stenosis; *LSS* lumbar spinal stenosis; *PDD* posterior direct decompression

**Fig. 3:**
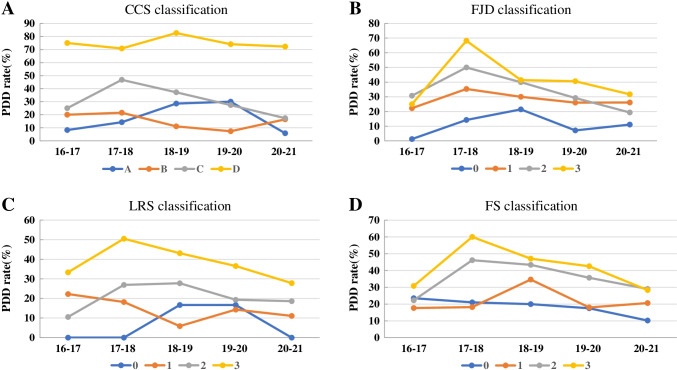
Annual posterior direct decompression (PDD) rate according to radiographic classification. **a** Central canal stenosis (CCS) classification; **b** facet joint degeneration (FJD) classification; **c** lateral recess stenosis (LRS) classification; **d** foraminal stenosis (FS) classification

The annual PDD rate for CCS grade C was seen to have increased in the second year (Table 2 and Fig. 3a). However, it then continued to decline to 17.39% in the most recent year, which was not significantly different from that of grade B (*p* = 1.00). The annual PDD rate for grade D remained at very high levels (70.88 ~ 82.61%) in the past five years of this study (Table 2 and Fig. 3a). We observed that the overall PDD rate for grade D is significantly larger than that of grade 3 FJD, LRS, and FS (Table 2). The overall PDD rate for grade 3 FJD, LRS, and FS was not significantly different in comparison to each other. According to diagnosis, the total PDD rate is 28.62% for LSS and 33.94% for LSS/spondylolisthesis (*p* = 0.12).

Univariate analysis showed year, CCS, FJD, LRS, and FS classification were all related with PDD, whereas sex, age, BMI, surgical segments, and spondylolisthesis were not. The PDD rate increased with the rise of all four parameter grade (*p* for trend < 0.05). Their overall and annual multivariable logistic regressions are shown in Table 3. Considering the overall PDD rate for grade A and B CCS is 15.02% (50/333), grade D CCS is the utmost risk factor for PDD (OR = 17.77, *p* < 0.001). The annual OR values for grade D CCS had consistently been high in all 5 years. Considering both the overall PDD rate for grades 0 and 1 of FJD, LSR, and FS is 25.87% (111/429), 11.93% (13/109), and 22.98% (108/470) and their overall OR values, we hypothesized that while they were all moderately increasing the risk of PDD, their effect on PDD is comparable.

**Table 3:** Multivariable logistic regression models for the associations between radiographic parameters and PDD

		Total OR (95%CI)	2016–2017 OR (95%CI)	2017–2018 OR (95%CI)	2018–2019 OR (95%CI)	2019–2020 OR (95%CI)	2020–2021 OR (95%CI)
CCS grade	A–B	Ref	Ref	Ref	Ref	Ref	Ref
	C	2.41 (1.67, 3.51)*	1.68 (0.41, 7.10)	3.31 (1.38, 8.56)	3.62 (1.66, 8.51)	3.06 (1.25, 8.38)	1.36 (0.68, 2.73)
	D	17.77 (10.54, 30.76)*	29.75 (2.23, 894.99)*	6.47 (1.95, 23.63)*	31.22 (8.80, 137.58)*	43.71 (12.36, 183.55)*	15.43 (6.29, 40.92)*
FJD grade	0–1	Ref	Ref	Ref	Ref	Ref	Ref
	2	1.24 (0.89, 1.73)	1.61 (0.32, 7.52)	1.59 (0.64, 3.99)	1.80 (0.88, 3.72)	1.61 (0.77, 3.42)	0.68 (0.35, 1.30)
	3	2.04 (1.32, 3.14)*	3.57 (0.34, 35.20)	3.63 (1.14, 12.55)	1.74 (0.64, 4.70)	3.17 (1.22, 8.34)	1.29 (0.54, 2.96)
LRS grade	0–1	Ref	Ref	Ref	Ref	Ref	Ref
	2	1.98 (1.05, 3.98)	0.76 (0.07, 8.37)	2.37 (0.43, 19.29)	3.15 (0.74, 21.91)	1.20 (0.36, 4.76)	2.20 (0.66, 10.09)
	3	4.63 (2.59, 8.92)*	3.54 (0.57, 32.98)	7.58 (1.75, 54.43)*	7.43 (1.94, 49.05)*	3.44 (1.18, 12.65)*	3.96 (1.29, 17.38)*
FS grade	0–1	Ref	Ref	Ref	Ref	Ref	Ref
	2	1.92 (1.36, 2.70)*	1.68 (0.33, 8.45)	1.71 (0.69, 4.26)	2.04 (1.00, 4.19)	3.20 (1.45, 7.26)*	1.99 (1.04, 3.83)*
	3	2.42 (1.64, 3.55)*	2.75 (0.46, 17.03)	2.66 (1.06, 6.91)*	2.58 (0.83, 8.09)	4.95 (2.11, 12.02)*	2.02 (0.94, 4.31)

*CCS* central canal stenosis; *FJD* facet joint degeneration; *LRS* lateral recess stenosis; *FS* foraminal stenosis; *OR (95%CI)* odds ratio (95% confidence interval); *Ref* reference; *statistical significance, *p* < 0.05

## Discussion

In the past four years after gaining further experience in LLIF surgery, our institution had observed a continuous decline in the annual staged surgery rates as well as annual PDD rates in grade 3 FJD, LRS, and FS, as well as grade C CCS segments. Despite this, the annual PDD rate for grade D remained at very high levels all through the 5 years (70.88 ~ 82.61%). Logistic regression analysis had shown grade D CCS as the utmost risk factor for PDD (OR = 17.77). And grade 3 LRS (OR = 4.63), grade 3 FS (OR = 2.42), grade C CCS (OR = 2.41), and grade 3 FJD (OR = 2.04) were also moderately correlated with PDD.

As an indirect decompression technique, many have concurred that severe CCS is a relative contradiction for LLIF [1, 2], and PDD is necessary for the presence of severe CCS. However, there are no clear definitions of severe CCS and no consensus on which degree of stenosis, where indirect decompression may not work. Several recent studies claimed that LLIF successfully achieves indirect decompression in patients with extremely severe CCS (Grade D) [9, 10, 28]. Shimizu et al. [9] reported that LLIF without PDD provided successful clinical outcomes for grade D patients at one year of follow-up, which is partly attributed to the resolution of pathological spinal instability. Likewise, Walker et al. [10] reported that LLIF may successfully achieve indirect decompression in grade D patients, if they have spondylolisthesis and a collapsed disc height. However, this study shows that the annual PDD rate for grade D CCS still remained at very high levels in the five years. Multivariate analysis showed grade D CCS as the utmost risk factor for PDD with the greatest OR value, which is consistent all throughout the five years of this study. In a radiographic comparative study [29], the mean central canal area (CCA) of grade D segments immediately after LLIF was 49.87 ± 18.81 mm^2^, which was significantly smaller than the mean pre-operative CCA of grade C segments (82.06 ± 26.97 mm^2^). Considering the indirect decompression mechanism of LLIF and the uncertainty of long-term clinical outcomes of LLIF for grade D, we recommend PDD for grade D patients. LLIF may also show success in selected grade D patients, who we are yet to accurately recognize based on our current knowledge.

Grade C segment accounts for 50.38% (454/901) of all our surgical segments. There is a continuous decline in the annual PDD rate for grade C segments in the past 4 years. In the last 12 months, only 17.39% of grade C segments needed PDD, which is not significantly different from grade B. Compared with the overall PDD rate of both grades A and B, the overall and the last year OR values of grade C were 2.41 (*p* < 0.05) and 1.36 (*p* = 0.39) respectively. Shimizu et al. [28] found that in the instance of severe stenosis, the mean CCA improved from 54.5 mm^2^ pre-operatively to 84.7 mm^2^ at three weeks post-operation, and to 132.6 mm^2^ at the last follow-up, an average 28.3 months later. Takahashi et al. [30] found that in spondylolisthesis patients with severe CCS (CCA ≤ 50 mm^2^), the mean CCA improved from 35.8 mm^2^ pre-operatively to 81.4 mm^2^ immediately after surgery, and to 105.7 mm^2^ at two years post-operatively. Indirect decompression produces immediate positive results that continued to improve over time as observed with the ligamentum flavum cross-sectional area and disc bulging both shrinking significantly [31]. Therefore, with this analysis, we can consider grade C stenosis as a proper indication for LLIF with low PDD risk.

Given that severe FJD may prevent distraction of the neural foramen during graft insertion, it has been originally considered as relative contraindications for LLIF [19, 20]. In our study, compared with the overall PDD rate of both grades 0 and 1 FJD, grade 3 FJD only moderately increased the risk of PDD (OR = 2.10, *p* = 0.001). Its OR value is only 1.29 (*p* = 0.56) in the most recent year. A study conducted by Navarro-Ramirez et al. [32] had found FJD and facet tropism were not associated with restoration of disc height, foraminal area, and CCA after LLIF, but instead, their study revealed significant clinical improvements were observed for LLIF patients with the presence of locked facets. Another study [33] claimed that the presence of metabolically active arthropathy had no significant effect on the amount of indirect decompression obtained with LLIF surgery. In fact, their data trends towards greater indirect decompression with degenerative facets. Our own study showed, with regard to the average change values of disc height, both sides foraminal height, canal diameter, and CCA, grade 3 FJD showed no significant differences compared with that of the other grades (data not published), unless it is completely fused. Thus, we consider severe FJD as a moderate risk factor for PDD.

This study showed that the overall OR value of grade 3 LRS is 4.83 (*p* = 0.04), compared with that of both grade 0 and 1 LRS. Narrowing of the lateral recess can cause radicular pain and/or low back pain. The diameter of the lateral recess increased bilaterally after LLIF [20]. Severe facet hypertrophy, synovial cysts, and osteophytes arising from the posterior endplates were reported as risk factors for lateral recess indirect decompression failure [20, 34]. While bony LRS may represent a risk factor for failure of indirect decompression [13, 16], we consider that LRS caused by soft tissue elements is not. MRI could not differentiate bony or non-bony LRS and grade 3 LRS segments account for 60.49% (545/901) of all segments; hence, we do not regularly see grade 3 LRS as a risk factor for PDD.

Indirect decompression via LLIF had shown to achieve great radiographic outcomes similar to direct approaches in terms of relieving FS [34]. In a prospective study, the foraminal area increased by 24.7% and the foraminal height by 13.5% after LLIF [20]. In a prospective randomized study [3], LLIF results in an increased foraminal area by 23% compared with 4.9% in MIS-TLIF on the approach side at three month follow-up. Kirnaz et al. [35] recommended indirect decompression for patients who have symptomatic FS as long as we can confirm the source of the pain by eliciting radicular symptoms with a Kemp’s test. However, at present, there has been no research that looks at the efficacy of LLIF for patients only with radiculopathy. Likewise, radiculopathy that prevailed over neurogenic claudication is not a good indication for LLIF in our institution. In our study, grade 3 FS moderately increases the risk of PDD (OR = 2.42, *p* < 0.001).

Our study had several limitations and can be identified as follows. Firstly, this was a retrospective study by nature. Secondly, the classification of CCS, LRS, and FS was based on MRI, which could not differentiate bony stenosis or soft tissue stenosis and could not fully distinguish the pathological features of stenosis. Thirdly, intra-operative and post-operative factors were not analyzed in the current study. Lastly, the current study does not involve symptom evaluations which in hindsight could have skewed some parts of our data. There is increasing evidence shown to pre-operative clinical symptoms as the main indicator of successful indirect decompression. Khalsa et al. [36] reported that pre-operative assessment of rest pain level in the supine position has a significant association with reduction in leg and back sores in patients undergoing indirect decompression. Two studies [19, 37] claimed that patients with radicular symptoms unimproved with flexion may require direct decompression, and it is proposed that if patients are able to achieve dynamic symptom relief in a sitting or lying position, they may benefit from LLIF without requiring direct decompression, which is consistent with our experience.

In conclusion, grade 3 FJD, LRS, and FS, as well as grade C CCS, moderately increase the risk of PDD, which means there are proper indications for LLIF with low PDD risk. Extreme severe CCS (grade D) is the greatest determinant to perform the PDD procedure after LLIF. We would regularly recommend PDD for grade D segments.

## Data Availability

The data will be available upon request.
